# Trends in Medicare Billing by Oncologists for Integrated Mental Health Care Services

**DOI:** 10.1001/jamanetworkopen.2026.0023

**Published:** 2026-02-25

**Authors:** Koral Blunt, Kevin Johns, Ann Scheck McAlearney, Lauren E. Miller

**Affiliations:** 1 Department of Otolaryngology–Head & Neck Surgery, The Ohio State University College of Medicine, Columbus; 2 Department of Psychiatry, The Ohio State University College of Medicine, Columbus; 3 Center for the Advancement of Team Science, Analytics, and Systems Thinking in Health Services and Implementation Science Research, Columbus, Ohio; 4 Department of Family and Community Medicine, The Ohio State University College of Medicine, Columbus

## Abstract

This cross-sectional study examines trends in Medicare billing by oncologists for integrated mental health care services, using the collaborative care model and the behavioral health integration model, from 2018 to 2024.

## Introduction

Depression is prevalent among patients with cancer, and US guidelines emphasize routine screening and coordinated, team-based care.^1^ The collaborative care model (CoCM) is an evidence-based approach to depression management that integrates the treating clinician, behavioral health manager, and psychiatric consultant to improve outcomes among patients with cancer.^2,3^ The behavioral health integration (BHI) model provides a related but less intensive framework to integrate behavioral health services. There are distinct reimbursement pathways for both models, but the extent to which oncologists bill for these services remains uncertain. We examined trends in Medicare billing by oncologists for CoCM and BHI services.

## Methods

We conducted a cross-sectional analysis of Medicare Physician/Supplier Procedure Summary (PSPS) files from 2018 to 2024, aggregating allowed services by Healthcare Common Procedure Coding System (HCPCS) code and clinician specialty. CoCM services were defined as codes 99492 to 99494 and G2214 (beginning in 2021). BHI services were defined as code 99484 (eTable 1 in Supplement 1). Oncology specialties (by Centers for Medicare & Medicaid Services code) included hematology/oncology (83), medical oncology (90), radiation oncology (91), surgical oncology (92), and gynecologic oncology (98). Outcomes included annual volume, oncologic adoption, and distribution across specialties. We used 2-sided Mann-Kendall tests to evaluate monotonic trends across years (α = .05). Analyses were performed in Python, version 3.11 (Python Software Foundation), using the pandas and matplotlib libraries. Because PSPS data are publicly available and deidentified, institutional review was not required per the Common Rule. This study followed the STROBE reporting guideline.

## Results

Between 2018 and 2024, clinicians billed 829 044 CoCM services and 1 356 006 BHI services (N = 2 185 050) to Medicare beneficiaries. During this period, utilization increased from 55 082 to 284 062 CoCM services and from 24 033 to 473 156 BHI services. Mann-Kendall tests confirmed increasing trends in total CoCM (τ = 0.71; *P* = .04), total BHI (τ = 1.00; *P* = .003), and oncology CoCM (τ = 0.71; *P* = .02) service counts; oncology BHI service counts (τ = 0.52; *P* = .12) and oncology share of BHI services (τ = −0.10; *P* = .88) did not increase (eTable 2 in Supplement 1). Oncology clinicians accounted for less than 1% each of CoCM and BHI services billed annually (Figure).

**Figure.**
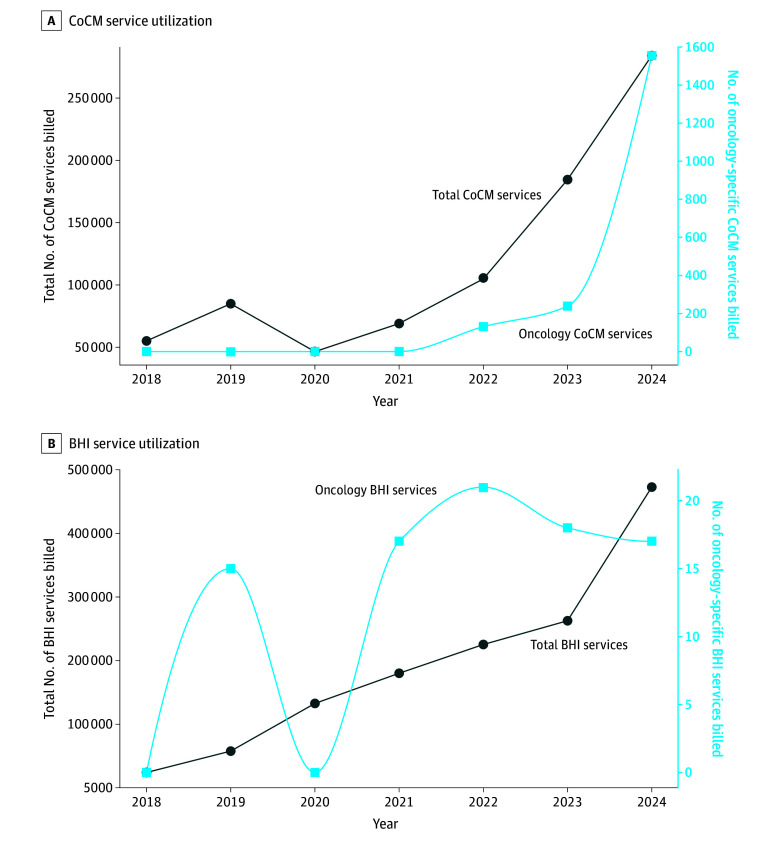
Collaborative Care Model (CoCM) and Behavioral Health Integration (BHI) Service Utilization in Medicare, 2018-2024 Total and oncology-specific CoCM services (A) and BHI services (B) billed annually. Oncology lines were drawn using a shape-preserving cubic spline for visual smoothing.

## Discussion

Despite robust evidence that integrated care improves depression outcomes,^4^ our findings suggest that 2018-2024 oncologist billing of CoCM or BHI services for Medicare beneficiaries remained scant. Several mechanisms likely contribute: the CoCM requires substantial infrastructure, which may be prohibitive for practices facing operational constraints; administrative complexity^5^ may limit implementation; alterative but less efficient referral pathways may predominate; and some health care systems may utilize grants or philanthropy to fund these services.

Study limitations include PSPS suppression of cells with 1 to 10 services, resulting in lower-bound national totals. Services could not be linked to unique clinicians or beneficiaries, so increases may reflect broader adoption, higher intensity among existing users, or changes in patient mix. Data from federally qualified health centers, Medicare Advantage, and commercial payers were excluded. Finally, specialty coding may misclassify multidisciplinary practices if claims were submitted under nononcology designations.

Depression and anxiety are prevalent in cancer care and worsen adherence, symptom burden, quality of life, and survival.^6^ Ambulatory cancer care is time-intensive, and competing appointments make separate mental health visits difficult during active treatment. Patients may not reliably access these services through primary care. CoCM and BHI services can be embedded in oncology practices to reach patients efficiently, support value-based oncology, and reduce disparities. Health systems should pilot oncology-specific models, such as centralized care managers and psychiatrists serving multiple clinics, integrating symptom tracking into routine workflows, and automating billing. Policy efforts could include risk-adjusted payment models and quality measures emphasizing sustained engagement.

In this cross-sectional study of Medicare claims, CoCM and BHI service utilization grew significantly from 2018 to 2024, but oncologist billing remained scant. Closing this gap is an opportunity to improve cancer care quality and patient well-being.

## Notes

Supplement 1. **eTable 1.** HCPCS Codes Used in Analysis **eTable 2.** Mann-Kendall Trend Tests for Annual Service Counts and Oncology Share, 2018-2024

Supplement 2.
Data Sharing Statement
