# Supplementary material for: Trends in Medicare Billing by Oncologists for Integrated Mental Health Care Services
Source: JAMA Netw Open. 2026 Feb 25;9(2):e260023. doi: 10.1001/jamanetworkopen.2026.0023 (PMC12936875; doi:10.1001/jamanetworkopen.2026.0023)
Supplement: Supplement 1. — eTable 1. HCPCS Codes Used in Analysis eTable 2. Mann-Kendall Trend Tests for Annual Service Counts and Oncology Share, 2018-2024 [file jamanetwopen-e260023-s001.pdf]

Supplemental Online Content

Blunt K, Johns K, McAlearney AS, Miller LE. Trends in Medicare billing by oncologists for integrated mental health care services. *JAMA Netw Open*. 2026;9(2):e260023. doi:10.1001/jamanetworkopen.2026.0023

eTable 1. HCPCS Codes Used in Analysis

eTable 2. Mann-Kendall Trend Tests for Annual Service Counts and Oncology Share, 2018-2024

This supplemental material has been provided by the authors to give readers additional information about their work.

© 2026 Blunt K et al. *JAMA Network Open*.

eTable 1. HCPCS Codes Used in Analysis

Billing Code	Description	wRVU
99492	Initial psychiatric collaborative care management, first 70 minutes in the first calendar month of behavioral health care manager activities, in consultation with a psychiatric consultant, and directed by the treating physician or other qualified health care professional, with the following required elements: outreach to and engagement in treatment of a patient directed by the treating physician or other qualified health care professional, initial assessment of the patient, including administration of validated rating scales, with the development of an individualized treatment plan, review by the psychiatric consultant with modifications of the plan if recommended, entering patient in a registry and tracking patient follow-up and progress using the registry, with appropriate documentation, and participation in weekly caseload consultation with the psychiatric consultant, and provision of brief interventions using evidence-based techniques such as behavioral activation, motivational interviewing, and other focused treatment strategies.	1.88
99493	Subsequent psychiatric collaborative care management, first 60 minutes in a subsequent month of behavioral health care manager activities, in consultation with a psychiatric consultant, and directed by the treating physician or other qualified health care professional, with the following required elements: tracking patient follow-up and progress using the registry, with appropriate documentation, participation in weekly caseload consultation with the psychiatric consultant, ongoing collaboration with and coordination of the patient's mental health care with the treating physician or other qualified health care professional and any other treating mental health providers, additional review of progress and recommendations for changes in treatment, as indicated, including medications, based on recommendations provided by the psychiatric consultant, provision of brief interventions using evidence-based techniques such as behavioral activation, motivational interviewing, and other focused treatment strategies, monitoring of patient outcomes using validated rating scales, and relapse prevention planning with patients as they achieve remission of symptoms and/or	2.05

© 2026 Blunt K et al. *JAMA Network Open*.

	other treatment goals and are prepared for discharge from active treatment.	
99494	Initial or subsequent psychiatric collaborative care management, each additional 30 minutes in a calendar month of behavioral health care manager activities, in consultation with a psychiatric consultant, and directed by the treating physician or other qualified health care professional	0.82
G2214	Initial or subsequent psychiatric collaborative care management, first 30 minutes in a month of behavioral health care manager activities, in consultation with a psychiatric consultant, and directed by the treating physician or other qualified health care professional	0.77
99484	Care management services for behavioral health conditions, at least 20 minutes of clinical staff time, directed by a physician or other qualified health care professional, per calendar month, with the following required elements: initial assessment or follow-up monitoring, including the use of applicable validated rating scales, behavioral health care planning in relation to behavioral/psychiatric health problems, including revision for patients who are not progressing or whose status changes, facilitating and coordinating treatment such as psychotherapy, pharmacotherapy, counseling and/or psychiatric consultation, and continuity of care with a designated member of the care team.	0.93

Abbreviations: BHI, Behavioral Health Integration; CoCM, Collaborative Care Model; HCPCS, Healthcare Common Procedure Coding System; wRVU, work relative value unit.

© 2026 Blunt K et al. *JAMA Network Open*.

eTable 2. Mann–Kendall Trend Tests for Annual Service Counts and Oncology Share, 2018-2024

Measure	Kendall τ	P value
Total CoCM services	0.71	.04
Oncology CoCM services	0.71	.02
Oncology share of CoCM services	0.71	.02
Total BHI services	1.00	.003
Oncology BHI services	0.52	.12
Oncology share of BHI services	-0.10	.88

Note: Two-sided Mann–Kendall tests evaluated monotonic trends across 2018–2024 (n=7 years).

© 2026 Blunt K et al. *JAMA Network Open*.
