# Supplementary material for: Trends in Medicare Billing by Oncologists for Integrated Mental Health Care Services
Source: JAMA Netw Open. 2026 Feb 25;9(2):e260023. doi: 10.1001/jamanetworkopen.2026.0023 (PMC12936875; doi:10.1001/jamanetworkopen.2026.0023)
Supplement: Supplement 2. — Data Sharing Statement [file jamanetwopen-e260023-s002.pdf]

Data Sharing Statement

Blunt. Trends in Medicare Billing by Oncologists for Integrated Mental Health Care Services. *JAMA Netw Open*. Published February 25, 2026. doi:10.1001/jamanetworkopen.2026.0023

Data
Data available: No

Additional Information
Explanation for why data not available: Data already publicly available
